# Mamba- and ResNet-Based Dual-Branch Network for Ultrasound Thyroid Nodule Segmentation

**DOI:** 10.3390/bioengineering11101047

**Published:** 2024-10-20

**Authors:** Min Hu, Yaorong Zhang, Huijun Xue, Hao Lv, Shipeng Han

**Affiliations:** 1 Department of Medical Electronics, School of Biomedical Engineering, Air Force Medical University, Xi’an 710032, China; hummmin@163.com (M.H.); zhangyaorong181@163.com (Y.Z.); xinyin20130419@163.com (H.X.); fmmulvhao@fmmu.edu.cn (H.L.); 2 School of Information and Control Engineering, Xi’an University of Architecture and Technology, Xi’an 710055, China

**Keywords:** thyroid nodule, visual Mamba, ResNet, ultrasound image segmentation

## Abstract

Accurate segmentation of thyroid nodules in ultrasound images is crucial for the diagnosis of thyroid cancer and preoperative planning. However, the segmentation of thyroid nodules is challenging due to their irregular shape, blurred boundary, and uneven echo texture. To address these challenges, a novel Mamba- and ResNet-based dual-branch network (MRDB) is proposed. Specifically, the visual state space block (VSSB) from Mamba and ResNet-34 are utilized to construct a dual encoder for extracting global semantics and local details, and establishing multi-dimensional feature connections. Meanwhile, an upsampling–convolution strategy is employed in the left decoder focusing on image size and detail reconstruction. A convolution–upsampling strategy is used in the right decoder to emphasize gradual feature refinement and recovery. To facilitate the interaction between local details and global context within the encoder and decoder, cross-skip connection is introduced. Additionally, a novel hybrid loss function is proposed to improve the boundary segmentation performance of thyroid nodules. Experimental results show that MRDB outperforms the state-of-the-art approaches with DSC of 90.02% and 80.6% on two public thyroid nodule datasets, TN3K and TNUI-2021, respectively. Furthermore, experiments on a third external dataset, DDTI, demonstrate that our method improves the DSC by 10.8% compared to baseline and exhibits good generalization to clinical small-scale thyroid nodule datasets. The proposed MRDB can effectively improve thyroid nodule segmentation accuracy and has great potential for clinical applications.

## 1. Introduction

Thyroid cancer is the most widespread type of cancer affecting the endocrine system. According to global cancer statistics from 2022, there were approximately 821,000 new cases of thyroid cancer, representing an increase of about 1.5 times compared to the incidence in 2020 [1,2]. Despite the relatively low overall mortality rate associated with thyroid cancer, the rise in incidence has led to a corresponding increase in related deaths. The condition not only impacts patients’ physical health but also threatens their overall quality of life and may leave lasting psychological effects. Fortunately, early detection and prompt treatment of thyroid nodules can greatly ease the progression of thyroid disease and prevent thyroid cancer. Traditionally, the diagnosis of thyroid nodules has relied heavily on the rich clinical experience of doctors. However, this method, which depends on empirical judgment, can be subjective, leading to inconsistencies in diagnosis accuracy. Additionally, it is both time-consuming and labor-intensive. With advancements in deep learning (DL), artificial-intelligence-assisted thyroid ultrasound diagnosis shows promise as a replacement for conventional diagnostic techniques, offering a potentially more convenient and precise approach.

However, existing DL methods still have some limitations in processing ultrasound images of thyroid nodules. The main reasons for this are that the inherent characteristics of ultrasound images result in a low contrast between the nodule boundary and the surrounding tissue, making it difficult for automatic segmentation algorithms to accurately capture the true boundary of the nodule, and noise and artifacts are often present in the ultrasound images, further increasing the difficulty of the segmentation task. In addition, the structural characteristics of thyroid nodules place higher demands on the generalization ability and robustness of the model. For example, in clinical practice, the size and shape of nodules vary greatly, ranging from tiny nodules to larger masses, which requires strong adaptability and robustness of the model. There is also the fact that the thyroid gland usually contains a variety of tissue structures, such as muscle, fat, and other glands, and this complex background information also interferes with the accurate segmentation of nodules. What is more, most nodules usually occupy only a very small portion of the entire image, which leads to category imbalance and makes the model prone to ignore small targets.

In order to improve the accuracy of thyroid nodule segmentation in ultrasound images, it is crucial to comprehensively consider the characteristics of ultrasound images and thyroid nodules. Above all, there is a need to enhance the model feature extraction capability, which is particularly capable of dealing with nodule boundaries and size variations in dimensions. To cope with these difficulties, we propose a novel Mamba- and ResNet-based dual-branch network (MRDB). The proposed method enhances the accuracy of thyroid nodule segmentation in ultrasound images by leveraging the strengths of both Mamba and CNNs. Specifically, it improves the model’s understanding of the global context while retaining the robust local feature extraction capabilities of CNNs. The MRDB model comprises three key components: a dual-encoder structure, a dual-decoder structure, and cross-skip connection linking the two branches. In the encoder, VSSB is integrated with ResNet-34 to capture multi-scale features of nodules and handle long-distance dependencies effectively. Meanwhile, the decoder employs distinct decoding strategies to accommodate both rapid image resolution recovery and detailed feature learning. Finally, cross-skip connection enables complementary feature integration between the different network branches. To summarize, the principal contributions of this research are outlined below:

- The Mamba- and ResNet-based dual-branching network (MRDB) aims to leverage Mamba’s capability for long-distance information modeling while combining the strong robustness and feature extraction abilities of ResNet to capture nodule features at multiple levels.
- A dual decoder is designed within the network. The left decoder focuses on the rapid recovery of image size while ensuring accurate and coherent details. The right decoder emphasizes progressively finer feature learning and recovery, aiming to capture and reconstruct image information from different perspectives.
- Cross-skip connection is introduced to facilitate feature interaction between the dual-branch network, enabling local detail features and global context information to complement each other and enhancing the model’s ability to capture boundaries and improve overall segmentation performance.
- A novel hybrid loss function is proposed, incorporating Smooth L1 loss into the traditional BCE and Dice loss to address issues of category imbalance and boundary concerns. Experimental results demonstrate that the MRDB achieves optimal segmentation performance.

## 2. Related Works

### 2.1. CNN-Based Segmentation Methods

CNNs represent a ubiquitous DL methodology within the field of computer vision, achieving notable successes in various applications of medical imaging. Particularly, CNNs have been highly effective in the segmentation of thyroid ultrasound images, marking one of the earliest successful applications of these networks in the medical domain. For example, the proposal of UNet [3] is an important landmark of DL in medical segmentation and a benchmark in the field of thyroid nodule segmentation. The success of this model has inspired numerous researchers to improve and extend it to develop multiple variants. Partial researchers have used prior knowledge to guide network learning to improve segmentation accuracy and robustness, including feature prior [4,5], shape prior [6,7,8,9], and so on. However, the validity of a priori knowledge is highly dependent on the quality of the training data, and improper a priori knowledge may amplify the errors present in the training data. Therefore, most of the studies focus on the improvement of the network structure to obtain higher accuracy improvement. These improvements include the use of convolutional variants to enhance the feature extraction capability of the model and expand the receptive field of the network. Specifically, improvements include replacing the basic block with multi-scale attentional convolution [10] or selective kernel convolution [11] in UNet, or introducing a stacked dilated convolution module [12,13,14,15,16] in the bottleneck of the network. However, for specific thyroid nodule segmentation tasks, blurred nodule boundaries in ultrasound images often pose significant challenges to the model. To overcome these challenges, researchers have adopted various strategies to improve the boundary segmentation capability of the model. EANet [17] proposed the edge attention preservation module so that the edge flow only processes information related to the boundary. The method based on boundary regression [18] uses the generated boundary heatmap as an additional supervised branch to reduce boundary segmentation errors. BFG&MSF-Net [19] captures edge details by designing a boundary feature guide module. Moreover, some researchers also use multi-branch networks to improve the segmentation ability of the model for nodules. MshNet [20] uses dual decoders to gradually segment nodules from coarse to fine. HNet [21] designs a dual-branch network based on CNN to realize the simultaneous learning of low-level detail and high-level semantic. Research on CNN-based thyroid nodule segmentation has made considerable progress. However, due to the specific spatial relationships between the morphology and location of thyroid nodules and the surrounding tissues, the local convolution operations of CNNs are insufficient for perceiving the pixel relationships over long-distance dependencies within the image. This limitation often results in inaccurate segmentation of thyroid nodules.

### 2.2. Transformer-Based Segmentation Methods

In contrast, a transformer [22] is capable of capturing the dependencies among each position in the input sequence through a self-attention mechanism. However, single-transformer-based models often lack spatial inductive bias when modeling local information. Therefore, integrating CNNs with transformers for thyroid nodule segmentation emerges as an effective approach. LCA-Net [23] designs contextual attention module based on the transformer. This module simultaneously utilizes the local feature information of CNN and the global feature advantage of transformer, which can effectively extract global context and local detail features. However, the effectiveness of the method in segmenting the boundary of insignificant nodules needs to be improved. Therefore, BTNet [24] improves the boundary attention mechanism so that it focuses more on the learning of boundary information to improve the segmentation accuracy of nodal boundaries. In addition, the boundary point supervision module designed by BPAT-UNet [25] enhances boundary features and generates desirable boundary points through a novel self-attentive pooling method to obtain more accurate boundary information. Trans-CEUS [26] combines a dynamic swin transformer into UNet to accurately segment thyroid nodules in contrast-enhanced ultrasound images. HEAT-Net [27] designs a channel-enhanced transformer to enhance the extraction of global features. SkaNet [28] combines convolutional features with self-attention mapping in the encoding stage, while using local and global information in thyroid nodule images to enhance feature discernment. Enhanced-TransUNet [29] builds enhanced transformer modules for improving the understanding of vector relations to realize remote connection. GLFNet [30] is designed to combine self-attention global–local fusion block, fusing global semantic information with local details. MLMSeg [31] proposes a multi-channel transformer module for capturing remote dependencies of global view between different nodes. In addition, Manh et al. [32] introduced the self-attention mechanism into adversarial learning to guide the segmentation model to learn lesion features from global features. This approach allows the model to learn more information about boundaries, shapes, and sizes, thus producing more reliable predictions. The fully supervised approach relies on manual labeling, and pixel-level labeling is a time-consuming and laborious process. Therefore, SABR-Net [33] uses a semi-supervised approach to design shadow-masked transformer to perceive the missing structures in the shadow region, which further improves the segmentation performance of the model in complex backgrounds. Transformer-based image segmentation algorithms model global information in the input sequence through the self-attention mechanism. While this approach addresses the issue of long-distance dependencies in the image segmentation process, it also introduces a higher computational cost in terms of floating-point operations. Consequently, this limitation restricts the further application of transformers in the medical field.

### 2.3. Mamba-Based Segmentation Methods

Mamba [34], with its unique state space models (SSMs), has demonstrated higher efficiency than transformers in processing long sequences. This makes Mamba a very promising choice for current applications. Ruan et al. [35] constructed the first pure Mamba-based medical segmentation model called VM-UNet. The model employs a visual state space block (VSSB) as a basic block for U-shaped networks to capture a wide range of contextual information. Du et al. [36] proposed a hybrid Mamba and CNN U-shaped network named MM-UNet for magnetic resonance (MR) images to achieve optimal segmentation of prostate in MR by designing a Mamba-based global-context-aware module with powerful remote modeling capabilities. However, standard convolution is usually used for feature extraction in CNNs, which has a fixed receptive field that makes it limited when dealing with deformed objects in images. Therefore, Zou et al. [37] utilized the synergistic effect of deformable convolution with specific dynamic receptive fields (DCN) and state space model (SSM) to design the DeMambaNet model. This enables the model to adapt to local structural deformations and spatial features in dental images, and improves the model’s adaptability and accuracy to overlapping structures and fine structures in dental X-ray images. Tang et al. [38] introduced a rotational Mamba-UNet for redundant structures in Mamba. This model solves the degradation problem of information transfer from shallow to deep networks by designing residual VSSB and rotated SSM. All these methods can effectively improve the segmentation performance in the corresponding medical tasks, but the introduction of complex modules will bring an increase of computational resource demand. In order to satisfy the requirement of device lightweighting, researchers have proposed several efficient network architectures in conjunction with the novel Mamba. Zhou et al. [39] proposed a lightweight and high-performance HL-UNet network. This network integrates the VSSB on a residual-enhanced adaptive attention module, which enables the model to utilize both global and local information for accurate heart segmentation. LightCF-Net [40] modeled remote spatial dependencies while maintaining high performance by adding a novel design of visually attentive Mamba modules to skip connections for modeling contextual dependencies and reducing background noise interference. Ultralight VM-UNet [41] achieves superior performance with the lowest computational complexity while keeping the total count of treatment channels unchanged. In addition, Ma et al. [42] proposed Semi-Mamba-UNet by integrating Mamba-based UNet and CNN-based UNet into a semi-supervised learning framework in order to address the high cost of expert annotation. The model is able to simultaneously generate pseudo-labels which cross-supervise each other at the pixel level, which enhances the model’s learning ability on unlabeled data. Mamba alleviates the modeling limitations of CNNs by employing a global receptive field and dynamic weighting, thereby offering advanced modeling capabilities comparable to those of transformers, without incurring the additional computational complexity typically associated with transformer models. These advantages make Mamba a current research hotspot and offer a novel solution for medical image segmentation.

## 3. Methods

**Figure 1:**
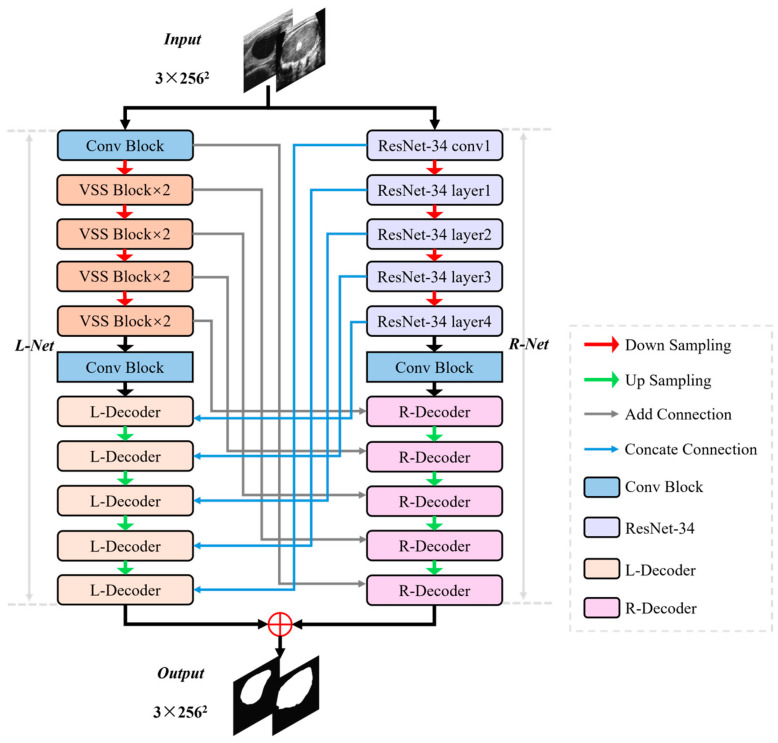
The overall framework of MRDB.

The MRDB network structure consists of three parts: a dual-branch encoder, a dual-branch decoder, and a cross-skip connection, as illustrated in Figure 1. Specifically, the encoder captures global and local features of the image using VSSB and ResNet-34, respectively. During the decoding stage, two distinct strategies are employed to efficiently recover image features from different perspectives. Finally, a multi-dimensional feature connection is established between the encoder and decoder via the cross-skip connection, thereby addressing nodule segmentation in complex scenes.

### 3.1. Dual-Encoder Branch

**Figure 2:**
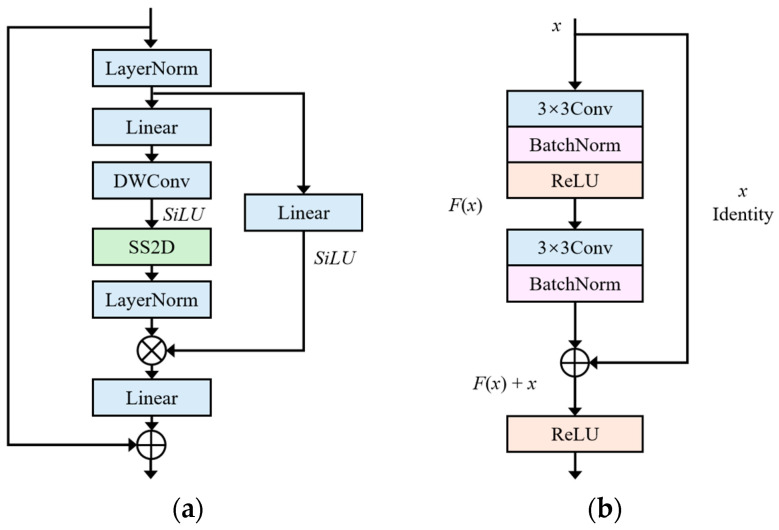
Main structure of dual-branch encoder. (**a**) VSSB; (**b**) ResNet-34.

The encoder is designed to extract richer feature representations using the advantages of VSSB and ResNet-34 in global receptive field and local feature processing. Its main structure is shown in Figure 2.

#### 3.1.1. Visual State Space Block

**Figure 3:**
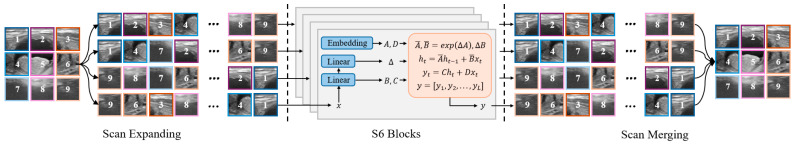
The 2D-selective-scan on an image.

The core VSSB of Mamba as one of the MRDB encoders aims to better capture the global contextual information of the image to compensate for the limitation of the localization of the convolutional operation. VSSB implements global receptive fields, dynamic selection of weights, and linear complexity, and uses 2D-selective-scan (SS2D) to selectively scan the input data. The detail structure of VSSB is shown in Figure 2a, and its core component SS2D is shown in Figure 3.

VSSB uses layer normalization on the input feature maps at first to ensure the stability of the data distribution. Subsequently, two parallel branches are used to process the feature. The first branch consists of a linear layer, depth-wise separable convolution, and an activation function that aims at digging into the deeper correlation of features. The features are then finely scanned and filtered by SS2D and normalized by layer normalization to further enhance feature differentiation and expressiveness. The second branch uses a simple linear layer and activation function to preserve the integrity of the original features. Subsequently, features originating from different paths are fused by element-wise multiplication to promote complementarity and integration of information. Finally, the fused features are added with the input features after a linear layer to form the output of VSSB.

SS2D is shown in Figure 3. The data transfer consists of three steps: scan expand, S6 block, and scan merge. The scan expand operation carries the input image through four paths to expand it into a sequence. Then, it is computed in parallel through S6 blocks. Subsequently, scan merging reconstructs and merges each sequence to form diverse features from different scan paths. Through a four-way scanning mechanism for spatial domain traversal, SS2D is able to bridge the discrepancy between the sequential structure of 1D selective scanning and the non-sequential structure of 2D visual data to help gather contextual information from different sources and perspectives.

#### 3.1.2. Residual Network

The amount of data is often small in thyroid nodule segmentation tasks, so choosing the right network is crucial. Residual networks enable better propagation of gradients during network training by introducing jump connections between convolutional layers so that the raw information from the input can be passed directly to the output layer. This mechanism effectively solves the problem of gradient vanishing in deep networks, allowing the model to maintain good performance during deep training. When working with small datasets, it is important to choose the right version of ResNet, because overly complex models can lead to overfitting. ResNet-34 has more layers than ResNet-18 and still maintains a relatively low number of parameters, which makes it more efficient in training and inference, while avoiding the risk of overfitting that comes with overly complex models. The depth of layer 34 is sufficient to capture more complex features without overfitting as easily as deeper models (such as ResNet-50, ResNet-101, or ResNet-152). Therefore, we adopt ResNet-34 as the second branch of MRDB in the encoder and utilize its local feature extraction capability to complement VSSB. The structure of ResNet-34 is shown in Figure 2b, and the module can be defined as:

(1) $$y=F\left(x,{\left\{W\right\}}_{i}\right)+x$$

where x and y are the input and output vectors for each layer, and the function $F\left(x,{\left\{W\right\}}_{i}\right)$ denotes the residual mapping to be learned. The F+x operation denotes connection and element-wise summation. As shown in Figure 2b, ResNet-34 has two convolutional layers, which can be expressed as $F=W_{2}\sigma \left(W_{1}x\right)$, where σ denotes ReLU. The details of ResNet-34 are presented in Table 1.

The introduction of residual block allows the network to learn the mapping more easily and maintains a stable training process even as the network layers deepen. When applying ResNet-34 as an encoder in MRDB, the final global average pooling layer and the layer fully connected to softmax are removed, and a sigmoid is set in the last convolutional layer for converting the feature map to a probability value between 0 and 1 to predict nodule and non-nodule regions.

### 3.2. Dual-Decoder Branch

**Figure 4:**
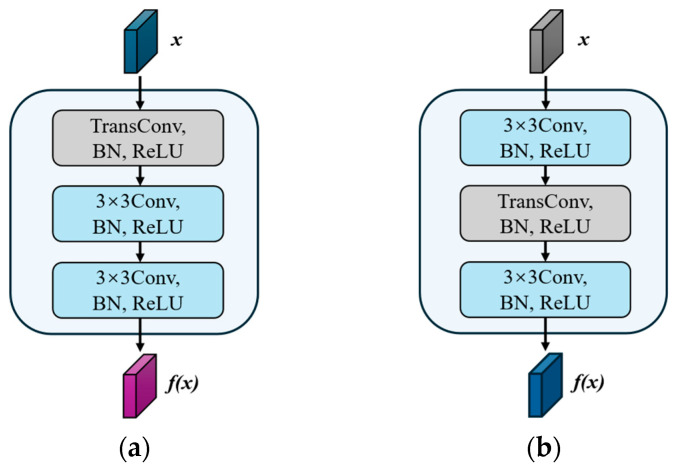
Main structure of dual-branch decoder. (**a**) Left decoder; (**b**) right decoder.

In the decoder, a transpose convolution with learnable parameters is used for upsampling instead of the interpolation method predefined in UNet. The decoder also employs a dual-branch structure with a different convolution strategy in each branch, and the complete structure is shown in Figure 4.

The design of the left decoder focuses on quickly recovering image resolution, meanwhile ensuring accuracy and coherence of image details. The resolution of the feature map is quickly restored by first performing an upsampling operation using the transpose convolution. Next, the sampled features are deeply learned and refined by two 3 × 3 convolutional layers. The feature map details are further learned and refined to ensure the accuracy and coherence of the image details.

The design of the right decoder focuses on progressively finer feature learning and recovery. Initial feature learning is first performed on the feature map output from the encoder through a 3 × 3 convolutional layer to capture and enhance local details. Subsequently, a transpose convolutional layer is used for upsampling to recover the feature map resolution without loss of information. Finally, another 3 × 3 convolution is used to further refine the features to ensure that the recovered image has clear details and complete structure.

The different designs of the dual decoders reflect the differential treatment of the feature recovery and learning process, aiming at capturing and reconstructing the image information from different perspectives.

### 3.3. Cross-Skip Connection

**Figure 5:**
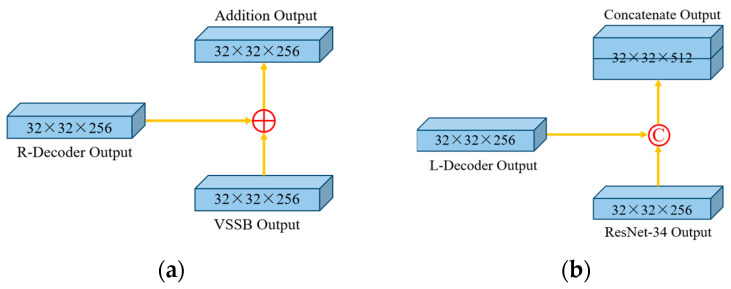
Schematic of concatenate and addition. (**a**) Addition; (**b**) concatenate.

The dual-branch network structure is designed to make the interaction between features richer and more flexible. Employing a single skip connection connected in UNet would limit the effective learning between different feature information. Therefore, an improved cross-skip connection is used for establishing a multi-dimensional feature mapping between the encoder and the decoder on both sides. For example, the feature map generated by the VSSB located on the left of the network is connected to the decoder on the right via feature addition, and the residual information is learned by adding the pixel values of the two feature maps together. The feature map generated by the right ResNet is concatenated with the decoder on the left to combine features from different sources through the feature information on the channel dimension. The decoder features generated by cross-skip connection can be defined as follows:

(2) $$X_{D}^{i}=f\left(x\right)=\left\{\begin{array}{cc} X_{E}^{i}⊕X_{D}^{n-i+1},\; & if\;X_{E}^{i}\in VSSB\\ Concat\left(X_{E}^{i},X_{D}^{n-i+1}\right),\; & if\;X_{E}^{i}\in ResNet \end{array}\right.$$

where, ⊕ represents feature addition, $Concat\left(\cdot \right)$ represents feature concatenate, $i\in \left(1,2,\cdots ,n\right)$ represents depth of encoder and decoder, $X_{E}^{i}$ represents the $i_{th}$ encoder, and $X_{D}^{i}$ represents the $i_{th}$ encoder. Concatenate and addition are shown in Figure 5.

### 3.4. Improved Loss Function

Binary cross-entropy (BCE) loss [43] and Dice loss [44] are two commonly used functions in image segmentation tasks, and they perform well in thyroid nodule segmentation. However, both approaches may expose certain limitations when dealing with small targets such as small thyroid nodules. Specifically, the BCE loss function measures performance of a model by calculating the difference between predicted probability distribution and true label. $L_{BCE}$ is defined as follows:

(3) $$L_{BCE}\left(p,g\right)=-\frac{1}{N}\left(\overset{N}{\underset{i=1}{\sum }}g_{i}\log\left(p_{i}\right)+\left(1-g_{i}\right)\log\left(1-p_{i}\right)\right)$$

where $p_{i}$ and $g_{i}$ represent the predicted value and the ground truth of pixel *i* in the input image, respectively. *N* is the total number of pixels. The difference value of all pixels in the image is calculated in BCE, which causes it to overemphasize the importance of background pixels and neglect the smaller but more important foreground pixels. However, in the thyroid nodule segmentation task, most of the lesion region accounted for a relatively small proportion of the entire image. Therefore, the introduction of Dice loss [45], which is more sensitive to unbalanced data, makes the model more focused on mining for thyroid nodule regions. $L_{Dice}$ is defined as follows:

(4) $$L_{Dice}\left(X,Y\right)=1-\frac{2\left|X\cap Y\right|}{\left|X\right|+\left|Y\right|}$$

where X and Y represent the predicted result and ground truth. Dice loss mitigates the problem of foreground and background imbalance in a sample by focusing primarily on the extent to which the segmentation result overlaps with the true label. Combining BCE and Dice loss can effectively mitigate the negative effects of category imbalance, but their insensitivity to border distributions may lead to irregular nodule segmentation profiles, thus affecting the accuracy of diagnostic results.

Therefore, we introduce the Smooth L1 loss [45] constrained model to learn the boundary information. $L_{Smooth_{L1}}$ is defined as follows:

(5) $$L_{Smooth_{L1}}\left(p,g\right)=f\left(x\right)=\left\{\begin{array}{c} 0.5{\left(p_{i}-g_{i}\right)}^{2},\;\;\left|p_{i}-g_{i}\right|<1\\ \left|p_{i}-g_{i}\right|0.5,\;\;otherwise \end{array}\right.$$

where $p_{i}$ and $g_{i}$ represent the predicted value and the ground truth of the nodule region in the input image, respectively. Smooth L1 constitutes a regression loss by calculating the difference between the predicted and labeled values of the nodule region and then summing these loss values. In contrast to BCE loss, Smooth L1 optimizes for outliers by separately constraining the foreground segmentation result to measure the difference value of the segmentation boundary. Specifically, the dynamic adaptability of Smooth L1 and its robustness to outliers allows the model to segment more accurate nodule segmentations by being smooth for large values of segmentation error and sparse for small errors.

Therefore, designing a hybrid loss that retains the superiorities of BCE, Dice, and Smooth L1 helps to overcome the limitations of a single loss function in some specific cases, to improving the overall performance and robustness. Specifically, the BCE loss is responsible for quantifying the difference between the predicted probability and the ground truth. Dice loss makes the model pay more attention to the similarity within the prediction region to alleviate the class imbalance problem. The Smooth L1 loss constrains the learning of the model with respect to the boundary. $L_{proposed}$ is defined as:

(6) $$L_{proposed}=L_{Dice}\left(Y_{p},Y_{g}\right)+L_{BCE}\left(Y_{p},Y_{g}\right)+0.5L_{Smooth_{L1}}\left(Y_{p},Y_{g}\right)$$

where $Y_{p}$ and $Y_{g}$ denote the segmentation result of the network and ground truth, respectively. The design model with this hybrid loss function is able to improve its sensitivity to boundaries while maintaining its sensitivity to small target detection. This method can not only improve the final segmentation accuracy, but also ensure the convergence of the model during the whole training.

## 4. Experiments and Results

### 4.1. Datasets

The performance of MRDB is evaluated on three publicly available thyroid nodule ultrasound datasets: TN3K [7,8], TNUI-2021, and DDTI [46]. Below are specific descriptions of these datasets.

TN3K: The dataset contains 3493 ultrasound images of thyroid nodules from 2421 patients, collected in Zhujiang Hospital of Southern Medical University. The nodules are manually marked by the sonographer. About 9% of ultrasound images have two or more thyroid nodules. The number of training and test images for the dataset are 2879 and 614, respectively. In addition, images from the same patient appear only in one subset when the dataset is divided.

TNUI-2021: The dataset consists of 1381 ultrasound thyroid nodule images. The images are obtained from 483 patients. They are acquired from two device species, Aixplorer and SAMSUNG WS80A, by doctors from Union Hospital of Fujian Medical University. The labeled images are labeled manually by expert pathologists at Union Hospital of Fujian Medical University. There are 966 training, 276 validation, and 139 testing thyroid nodule ultrasound images.

DDTI: The dataset contains 637 thyroid images taken from a single ultrasound device, each accurately labeled with information about a single nodule. The DDTI dataset is designated as an external test set due to its small amount of data, and is used to measure the algorithm’s ability to generalize across datasets. Specifically, after the model completes training on the TN3K dataset, it is evaluated on the DDTI dataset. In this way, the diagnostic accuracy and adaptability of the model on unseen data is verified.

### 4.2. Experiment Settings

All experiments have been performed on NVIDIA GeForce RTX 4090 GPU, Pytorch 2.0, and CUDA 11.8. The Adam optimizer is used in the training phase, the initial learning rate is 0.001, the size of the input image is 256 × 256, the batch size is 32, and the model is trained with 200 epochs. In addition, various data augmentation techniques are applied in the training phase to further improve the robustness of the model, such as randomly adding noise, histogram equalization, color gamut change, rectangle dropping, and pixel enhancement.

### 4.3. Evaluation Metrics

In order to evaluate the effectiveness of the model, the common evaluation metrics Dice similarity coefficient (DSC), Jaccard, sensitivity, 95% Hausdorff distance (HD95), and false negative rate (FNR) [47,48] are used to quantify the segmentation performance of models.

DSC is a metric that measures the degree of overlap between two sets of samples and is particularly suitable for medical image segmentation. A high DSC value indicates that the segmentation result is highly consistent with the true label, which is crucial for disease diagnosis and treatment planning. The definition is as follows:

(7) $$DSC\left(A,B\right)=2\frac{\left|A\cap B\right|}{\left|A\right|+\left|B\right|}$$

Jaccard is another commonly used overlap metric that calculates the ratio of the intersection of two sets to their union. Jaccard can provide additional information about the integrity of segmentation results in medical image segmentation, especially when dealing with complex or irregularly shaped lesions. The definition is as follows:

(8) $$Jaccard\left(A,B\right)=\frac{\left|A\cap B\right|}{\left|A\cup B\right|}$$

Sensitivity measures the ability to correctly identify positive cases. This metric is critical in medical diagnostics for assessing the reliability and safety of algorithms. The definition is as follows:

(9) $$Sensitivity=\frac{TP}{TP+FN}$$

HD95 is used to measure the maximum distance between two-point sets and can be used to assess the consistency of the segmentation boundaries of thyroid nodules. The definition is as follows:

(10) $$HD95=\underset{k95\%}{\max}\left[d\left(A,B\right),d\left(B,A\right)\right]$$

FNR is a very important indicator in medical diagnosis, reflecting the proportion of diagnoses that fail to correctly identify the actual sick individual. Since the number of positive samples (nodules) in thyroid nodule images is usually much less than the number of negative samples (non-nodule regions), FNR can more accurately reflect the performance of the model in detecting positive samples. The definition is as follows:

(11) $$FNR=\frac{FN}{TP+FN}$$

where *A* represents the labeled thyroid nodule region, B represents the thyroid nodule region segmented by the algorithm, and TP and FN denote true positive and false negative in image processing, respectively.

### 4.4. Comparison with Different Methods

**Figure 6:**
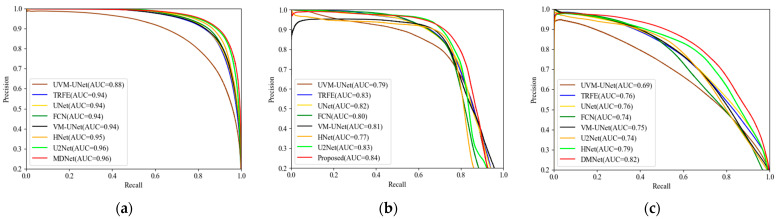
PR curves of different advanced models on three datasets. (**a**) TN3K; (**b**) TNUI-2021; (**c**) DDTI.

The proposed MRDB is compared with advanced medical segmentation methods including UVM-UNet [41], TRFE [7], UNet [3], FCN [49], VM-UNet [35], HNet [21], and U2Net [50] on TN3K, TNUI-2021, and DDTI datasets. To show the performance of the proposed method visually, a comparison of the precision–recall (PR) curves of MRDB and SOTA on the three datasets is shown in Figure 6. The PR curve provides a more comprehensive evaluation of model performance by focusing on the accuracy and coverage of the model in positive categories. It can be seen that MRDB achieved the highest PR-AUC of 0.97, 0.84, and 0.82 on the three datasets, respectively. In addition, MRDB maintains a high precision rate throughout the recall range. This shows that our method has stronger robustness and accuracy in dealing with complex data.

#### 4.4.1. Comparison on TN3K Dataset

The comparison results on the TN3K dataset are shown in Table 2, where MRDB achieves the highest accuracy rates of 0.9002, 0.8185, and 0.8911 for DSC, Jaccard, and sensitivity, respectively. Compared with the baseline model UNet, DSC increases from 0.8622 to 0.9002, an improvement of 3.8%. Jaccard increases from 0.7578 to 0.8185, an improvement of 6.07%. On HD95, a metric used to assess boundary fit, MRDB achieved the optimal segmentation results, decreasing from 16.0933 to 10.3465 compared to UNet. Moreover, MRDB is 1.1% higher in DSC, 1.8% higher in Jaccard, and 1.7675 lower in HD95 compared to U2Net. The superiority of MRDB in boundary segmentation is proved.

**Figure 7:**
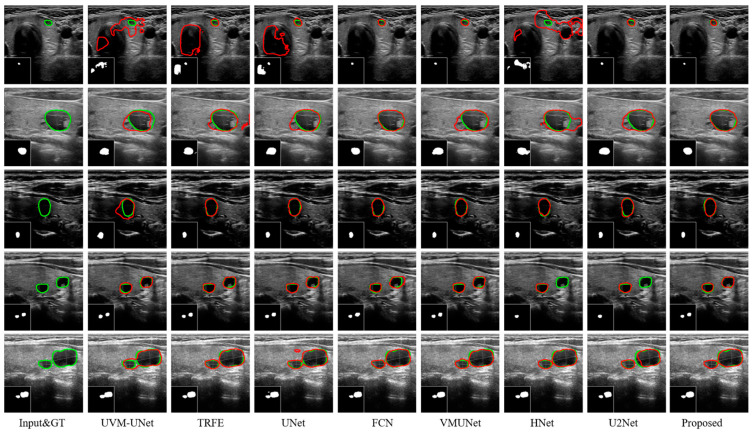
Segmentation results achieved by eight distinct methods on the TN3K dataset. The green and red lines represent ground truth and segmentation results, respectively.

Figure 7 provides the segmentation results of MRDB and other advanced methods on the TN3K dataset. The first column is the input image, and the corresponding ground truth is marked with a green line. The second to ninth columns show the segmentation results of UVM-UNet, TRFE, UNet, FCN, VM-UNet, HNet, U2Net, and the proposed method, respectively, where the red lines represent the model segmentation results. From the segmentation results, existing methods produce varying degrees of over-segmentation and under-segmentation for nodules with fuzzy boundaries of different sizes in the first three rows. For example, UVM-UNet, TRFE, UNet, and HNet misclassified the black area as a nodule when processing the small nodule in the first row, resulting in a segmentation with multiple nodules. In the complex case species containing multiple nodules in the third and fourth rows, HNet and U2Net tend to underestimate the actual extent of the nodules and exhibit under-segmentation. In contrast, models such as UVM-UNet and UNet incorrectly classify some non-nodule regions into the range of nodules, resulting in over-segmentation. Compared with other methods, MRDB shows significant advantages in dealing with the above situation. It is able to outline nodule boundaries that are close to the ground truth and outperforms other methods in terms of accuracy and fineness of multiple nodule segmentation.

#### 4.4.2. Comparison on TNUI-2021 Dataset

The MRDB model is further experimentally validated on the TNUI-2021 dataset, and the results are shown in Table 3. MRDB achieves an accuracy of 0.8060, 0.6750, and 0.7452 in DSC, Jaccard, and sensitivity, respectively. Compared with the base structure UNet, it improves 3.1%, 4.24% and 0.39%, respectively. The HD95 is only 7.0530, which is 2.5971 lower than with U2Net, achieving the current optimal segmentation performance.

**Figure 8:**
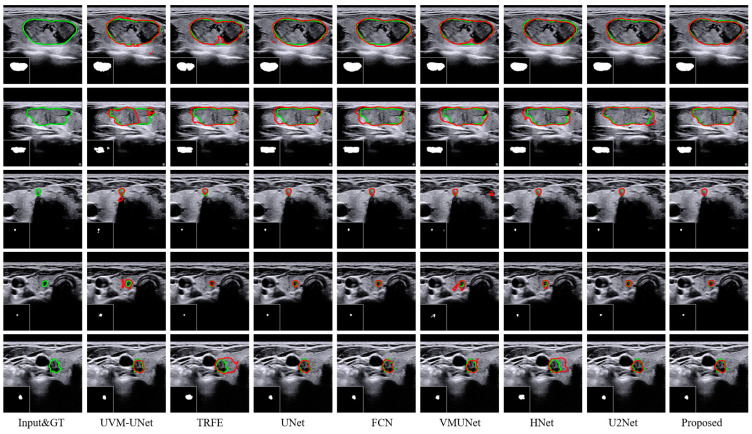
Segmentation results achieved by eight distinct methods on the TNUI-2021 dataset. The green and red lines represent ground truth and segmentation results, respectively.

Figure 8 provides a visual comparison of the segmentation results obtained by the MRDB method and other state-of-the-art techniques on the TNUI-2021 dataset. The ability of each model to handle different sizes and shapes of nodules is presented visually. When dealing with nodules with irregular edges in the first and second rows, the segmentation boundaries of MRDB are closer to the true label than other methods. In the segmentation of small-sized nodules in the second to fourth rows, models such as UVM-UNet, TRFE, and UNet suffer from over-segmentation. In contrast, the segmentation results of MRDB are highly consistent with the ground truth. This indicates that MRDB has higher robustness and accuracy in dealing with complex situations. By quantitative analysis and visualization of the results, MRDB demonstrates the best accuracy and excellent segmentation performance.

#### 4.4.3. Generalization on DDTI Dataset

To evaluate the generalization capability of the MRDB method, the trained model is tested on the DDTI dataset as an external validation set. The results are shown in Table 4. MRDB achieves the highest segmentation accuracy on the DDTI dataset, and its segmentation accuracy is much higher than that of HNet and U2Net, both of which have comparable segmentation accuracies to MRDB on TN3K and TNUI-2021. Compared to U2Net, MRDB has improved DSC by 3.61%, Jaccard by 4.49% and sensitivity by 8.73%. In addition, the boundary segmentation error is reduced by 3.5665, and FNR is reduced by 0.873.

**Figure 9:**
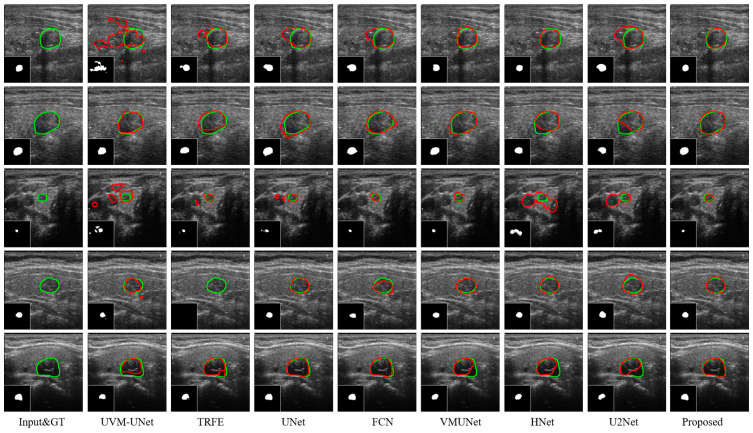
Segmentation results achieved by eight distinct methods on the DDTI dataset. The green and red lines represent ground truth and segmentation results, respectively.

The visualization of the thyroid segmentation methods on the DDTI dataset is shown in Figure 9. By comparing the results, we can see that the segmentation accuracy of MRDB for nodules is better than other methods such as UVM-UNet and TRFE, as it reduces over-segmentation and under-segmentation. Existing methods may not cover the nodule region comprehensively, or incorrectly include non-nodule regions in the nodule range. MRDB can maintain high segmentation accuracy and completeness in different situations, effectively avoiding the above problems. It shows excellent performance in handling unseen data.

### 4.5. Ablation Study

In order to evaluate the effectiveness of each enhancement in the proposed MRDB model, ablation studies are systematically performed on both the network components and the loss function.

#### 4.5.1. Network Components

The ablation experiments of the designed dual-branch network, encoder and decoder block, and cross-skip connection (CSC) are conducted and analyzed. The impact of each improvement on MRDB performance is fully verified. The results are shown in Table 5.

Effect of dual UNet in the MRDB: The impact on network performance of using a single UNet to implement the dual encoder–decoder structure is compared. As shown in the second row, compared with the baseline model UNet, the DSC improves by 1.45%, Jaccard increases by 2.26%, and HD95 decreases by 0.5432 with the dual encoder–decoder structure. The results show that the dual encoder–decoder structure can effectively improve the segmentation accuracy.

Effect of CSC in the MRDB: In order to efficiently transfer information between encoders and decoders on both sides, CSC is introduced. Both concatenate and addition interactions are used to increase the diversity of features and effectively convey key information. As shown in the third row, the model with CSC has better segmentation performance compared to the method without CSC. This indicates that CSC is more conducive to feature fusion in MRDB compared to the original skip connection.

Effect of ResNet-34 in the MRDB: ResNet-34 is used as a unilateral encoder in order to enhance the feature extraction capability of the model. The deep features are captured by the powerful feature extraction capability. As shown in the fourth row, the DSC and Jaccard of the model with ResNet-34 are improved by 1.02% and 1.63%, respectively, and the HD95 is reduced by 2.0788 compared to the unused method. The results show that the combination with ResNet-34 achieves the accurate capture of deep features.

Effect of R-Decoder in the MRDB: A convolution–upsampling–convolution strategy is used in the right decoder, focusing on progressively finer feature learning and recovery. As shown in the fifth row, the model with the improved decoder structure improves the DSC and Jaccard by 0.56% and 0.65%, respectively, and reduces the HD95 by 0.9065. The results show that the dual decoder using different decoding methods helps to achieve richer and more flexible feature interactions.

Effect of VSSB in the MRDB: In order to alleviate the problem of weak perception of long-distance pixel relationship by convolutional neural network, VSSB is introduced in another encoder to capture the contextual information. As shown in the last row, the complete MRDB with VSSB achieves the highest DSC value of 90.02% and Jaccard value of 81.85%, and the HD95 is reduced to 10.3465. The results show that the introduction of VSSB efficiently models the long-distance information in order to improve the segmentation accuracy of the model.

#### 4.5.2. Different Loss Function

In the process of model training, the choice of loss function is crucial. Table 6 shows the performance impact of different loss functions on MRDB in the thyroid segmentation task. The commonly used Dice loss, BCE loss, and their combinations are compared with our proposed hybrid loss function.

Dice Loss: Dice loss is widely used in image segmentation tasks, aiming to maximize the Dice similarity coefficient between the predicted results and the real labels. The experimental results show that the MRDB model with Dice loss achieves a DSC of 0.8788, a Jaccard index of 0.7839, a sensitivity of 0.8741, an HD95 of 12.3978, and an FNR of 0.1259 on the TN3K dataset.

BCE Loss: BCE loss is another commonly used loss function, mainly for binary classification problems. When the BCE loss is applied in the MRDB model, the model has a DSC of 0.8861, a Jaccard of 0.7955, a sensitivity of 0.8483, an HD95 of 11.5968, and an FNR of 0.1517 on the TN3K dataset. BCE loss is more favorable for segmentation boundary accuracy than Dice loss, but underperforms in sensitivity and FNR.

Combined Loss: The combination of BCE and Dice forms a composite loss function that aims to synthesize the advantages of both. This is a common strategy currently used to optimize segmentation models. The MRDB model using the composite loss function has a DSC of 0.8882, a Jaccard of 0.7988, a sensitivity of 0.8744, an HD95 of 12.1296, and an FNR of 0.1256 on the TN3K dataset. It shows that the combined loss can better balance the fine segmentation of the boundary while improving the segmentation accuracy of the model.

Proposed Loss: Finally, we tried the innovative combined loss function combining BCE, Dice, and Smooth L1. Experimentally, the MRDB model using the proposed loss has a DSC of 0.9002, a Jaccard of 0.8185, and a sensitivity of 0.8911 on the TN3K dataset, whereas the HD95 and the FNR are reduced to 10.3465 and 0.1089, respectively. Thus, the proposed loss function not only improves the overall segmentation performance of the model, but also achieves significant improvement in boundary refinement and localization accuracy.

## 5. Discussion

Due to the variable size and blurred edges of thyroid nodules in ultrasound images, accurate segmentation has become a significant challenge. Although existing methods have made substantial improvements in segmentation performance for this task, various limitations remain. Specifically, most methods struggle to accurately capture the true boundaries of nodules when the edges are blurred, and they tend to over-segment or under-segment when dealing with nodules of different sizes. Therefore, we propose a Mamba- and ResNet-based dual-branch network (MRDB) to enhance the accuracy and robustness of the model for challenging nodule segmentation tasks.

Global features in ultrasound images of thyroid nodules provide crucial information about the surrounding tissues and organs, which is essential for understanding the developmental status and potential impact of the nodule. The MRDB network extracts both global and local features during the encoding stage, allowing for complementary and coordinated information capture. This comprehensive approach enables the model to fully understand the nodule and its environment. In contrast, U2Net focuses on multi-level feature extraction and fusion but does not sufficiently prioritize global information in the initial stages. This may affect the final segmentation performance when dealing with images in complex backgrounds. Similarly, HNet utilizes a dual-branch structure aimed at learning both low-level details and high-level semantics. However, the feature fusion module designed by HNet at the bottleneck layer includes too many convolutional layers, which might result in information loss and hinder subsequent feature recovery and interaction. The integration of global and local features in MRDB facilitates a better understanding of the developmental status and potential impact of nodules when processing ultrasound images of thyroid nodules, thereby providing more reliable support for clinical diagnosis.

To further optimize the segmentation performance of the model, we employ a hybrid loss function that combines BCE loss, Dice loss, and Smooth L1 loss. The limitations of a single loss function in a given situation can be overcome by using it in combination to improve the overall performance and robustness of the model. Specifically, BCE loss evaluates model performance by calculating the difference between the predicted probability distribution and the true label, and it excels in classification accuracy at the pixel level. However, BCE loss may overemphasize the importance of background pixels, which is particularly detrimental when dealing with the thyroid nodule segmentation task. Dice loss, on the other hand, measures the degree of overlap between the predicted segmentation results and the true labels, allowing the model to focus more on the detection of nodal regions, which is particularly suitable for dealing with the problem of category imbalance. However, Dice loss does not perform well in dealing with boundary blurring and may result in inaccurate segmentation boundaries. Smooth L1 loss constitutes a regression loss by calculating the difference between the predicted and true values, and is able to optimize the foreground segmentation results, especially the accuracy of the segmentation boundaries. Dynamic adaptation and smoothing of large errors allow it to perform well in boundary learning. Therefore, by combining BCE loss, Dice loss, and Smooth L1 loss, our model is able to improve classification accuracy, alleviate the category imbalance problem, and optimize boundary learning for more accurate segmentation results.

Improving the lightweight performance of the model to meet the requirements of embedded and mobile devices remains a major challenge for clinical applications. Therefore, future research directions will focus on enhancing the operational efficiency of the model to ensure that it can perform efficient and accurate segmentation tasks even in resource-constrained environments. We aim to reduce redundant computations through in-depth research on model compression techniques and parameter sharing strategies, thereby improving the generalizability and efficiency of the models to better serve clinical practice.

## 6. Conclusions

This paper proposes a novel Mamba- and ResNet-based dual-branch network (MRDB) for accurate segmentation of thyroid nodules in ultrasound images, particularly focusing on small-sized or irregularly shaped nodules. The MRDB network primarily consists of a dual encoder for acquiring local and global features, a dual decoder for reconstructing image information from multiple perspectives, and cross-skip connection for facilitating feature interaction. Additionally, a hybrid loss function is designed for model training. Experiments conducted on three publicly available thyroid nodule ultrasound image datasets show that MRDB achieves the best segmentation accuracy and optimal performance on an external dataset. This indicates that MRDB has strong generalization capabilities and significant potential for clinical applications.

## Figures and Tables

**Table 1:** Architecture of ResNet-34. Building layers are shown in brackets (shown in Figure 2b) and the numbers of blocks stacked.

Layer Name	Output Size	34-Layer
conv 1	128 × 128	7 × 7, 64, stride 2
Layer 1	64 × 64	3 × 3 max pool, stride 2
		$\left[\begin{array}{c} 3\times 3,64\\ 3\times 3,64 \end{array}\right]\times 3$
Layer 2	32 × 32	$\left[\begin{array}{c} 3\times 3,128\\ 3\times 3,128 \end{array}\right]\times 4$
Layer 3	16 × 16	$\left[\begin{array}{c} 3\times 3,256\\ 3\times 3,256 \end{array}\right]\times 6$
Layer 4	8 × 8	$\left[\begin{array}{c} 3\times 3,512\\ 3\times 3,512 \end{array}\right]\times 3$

**Table 2:** Comparison results on TN3K.

Method	DSC	Jaccard	Sensitivity	HD95	FNR
UVM-UNet	0.7962	0.6614	0.7709	21.9785	0.2291
TRFE	0.8651	0.7622	0.8692	17.7802	0.1308
UNet	0.8622	0.7578	0.8867	16.0933	0.1133
FCN	0.8736	0.7755	0.8549	13.7297	0.1451
VM-UNet	0.8684	0.7674	0.8606	14.4468	0.1451
HNet	0.8830	0.7906	0.8764	14.1400	0.1236
U2Net	0.8892	0.8005	0.8906	12.1140	**0.1023**
Proposed	**0.9002**	**0.8185**	**0.8911**	**10.3465**	0.1089

Bold represents the best results.

**Table 3:** Comparison results on TNUI-2021.

Method	DSC	Jaccard	Sensitivity	HD95	FNR
UVM-UNet	0.7495	0.5994	0.7048	11.9571	0.2952
TRFE	0.7753	0.6331	0.7232	11.0230	0.2768
UNet	0.7750	0.6326	0.7413	9.6501	0.2587
FCN	0.7680	0.6234	0.6871	7.6442	0.3129
VM-UNet	0.7797	0.6389	0.7206	10.1937	0.2794
HNet	0.7874	0.6494	0.7217	8.4846	0.2783
U2Net	0.7945	0.6590	0.7369	9.5981	0.2631
Proposed	**0.8060**	**0.6750**	**0.7452**	**7.0530**	**0.2548**

Bold represents the best results.

**Table 4:** Generalization results on DDTI.

Method	DSC	Jaccard	Sensitivity	HD95	FNR
UVM-UNet	0.5052	0.3380	0.3663	40.1927	0.6337
TRFE	0.6522	0.4839	0.5494	29.3721	0.4506
UNet	0.6416	0.4723	0.5249	26.8659	0.4751
FCN	0.6378	0.4682	0.5262	31.1645	0.4738
VM-UNet	0.6626	0.4954	0.5697	30.2809	0.4303
HNet	0.7115	0.5522	0.6345	28.1954	0.3655
U2Net	0.7135	0.5546	0.6496	28.2147	0.3504
Proposed	**0.7496**	**0.5995**	**0.7369**	**24.6482**	**0.2631**

Bold represents the best results.

**Table 5:** Ablation Study.

Base	Dual UNet	CSC	ResNet-34	R-Decoder	VSSB	DSC	Jaccard	HD95
√						0.8622	0.7578	16.0933
√	√					0.8767	0.7804	15.5501
√	√	√				0.8804	0.7864	15.2983
√	√	√	√			0.8906	0.8027	13.2195
√	√	√	√	√		0.8962	0.8120	12.3130
√	√	√	√	√	√	**0.9002**	**0.8185**	**10.3465**

Bold represents the best results.

**Table 6:** Loss function.

Loss Function	DSC	Jaccard	Sensitivity	HD95	FNR
*L_Dice_*	0.8788	0.7839	0.8741	12.3978	0.1259
*L_BCE_*	0.8861	0.7955	0.8483	11.5968	0.1517
*L_Combined_*	0.8882	0.7988	0.8744	12.1296	0.1256
*L_Proposed_*	**0.9002**	**0.8185**	**0.8911**	**10.3465**	**0.1089**

Bold represents the best results.

## Data Availability

The TN3K dataset used in the experiments can be found at https://233github.com/haifangong/TRFE-Net-for-thyroid-nodule-segmentation/tree/main/picture (accessed on 8 May 2024), the TNUI-2021 dataset can be found at https://github.com/zxg3017/TNUI-2021- (accessed on 8 May 2024), and the DDTI dataset can be found at https://www.kaggle.com/datasets/dasmehdixtr/ddti-thyroid-ultrasound-images/data (accessed on 8 May 2024).
